# Ninety-Day Emergency Department Rebound Following Adult Tonsillectomy: A Retrospective Cohort Study

**DOI:** 10.1177/19160216251333350

**Published:** 2025-05-14

**Authors:** Kalpesh Hathi, Gizelle Francis, JoAnne Douglas, Evan Nemeth, Paul Hong

**Affiliations:** 1 Division of Otolaryngology—Head and Neck Surgery, Department of Surgery, Dalhousie University, Halifax, NS, Canada; 2 Faculty of Medicine, Dalhousie University, Halifax, NS, Canada; 3 Department of Surgery, Dalhousie University, Halifax, NS, Canada; 4 Performance and Analytics, Quality and System Performance, Nova Scotia Health, Halifax, NS, Canada

**Keywords:** tonsillectomy, complications, pain, bleeding, emergency department, rebound

## Abstract

**Importance:**

Post-tonsillectomy complications often present in emergency departments (EDs). Reducing postoperative ED visits is one strategy to relieve the strain on healthcare systems and patients.

**Objective:**

To assess the rate and reason for ED visits within 90-days post-discharge from adult tonsillectomy.

**Design:**

Retrospective cohort study.

**Setting:**

Nova Scotia, Canada.

**Participants:**

All adult patients (≥16 years old) with a Nova Scotia Healthcare card who underwent a tonsillectomy in Nova Scotia, Central Zone from April 1, 2016 to March 31, 2022, and had an ED visit anywhere in Nova Scotia from April 1, 2016 to June 30, 2022, to allow a 90-days post-discharge window.

**Methods:**

Retrospective chart review utilizing administrative datasets for province-wide ED visits within 90-days post-discharge from an adult tonsillectomy. The patients’ first ED visit postoperation was analyzed.

**Results:**

Overall, 356 adult patients underwent tonsillectomy, of which 129 (36.2%) presented to the ED within 90 days. Of these, 99 were related to the tonsillectomy, resulting in a surgery-specific ED rebound rate of 27.8%. Most surgical ED visits (84/99, 84.8%) occurred within 7 days, most commonly for bleeding (47/99, 47.5%) and pain (36/99, 36.4%). Of the surgical visits, 26/99 (26.3%) were admitted, with 22/26 (84.6%) for bleeding. Of the surgical visits not related to bleeding, 48/52 (92.3%) were discharged home or left without being seen, which suggests 48/99 (48.5%) surgical ED visits may be preventable.

**Conclusion:**

The ED rebound rate for visits related to the tonsillectomy was 27.8% in our population. Given the potentially severe consequences of post-tonsillectomy bleeding, a high ED visit rate may be necessary. However, optimization of postoperative pain control along with greater access to urgent outpatient otolaryngology and primary care resources may reduce the burden of ED visits. This data adds to recent literature suggesting a higher rate of healthcare usage post-adult tonsillectomy.

## Graphical abstract

**Graphical Abstract:**
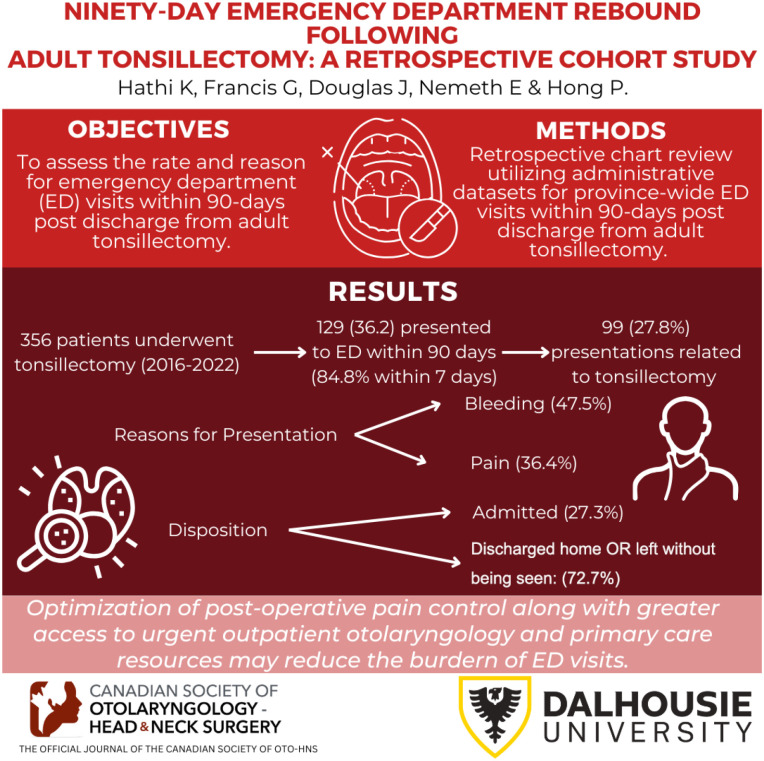

## Key Messages

1. The rate of emergency department usage after adult tonsillectomy is >20%, which is higher than previously expected.
2. Half of these visits may be preventable with improved patient education and pain management.
3. These results may help guide patient education and management, as well as future policy and resource allocation.

## Introduction

Tonsillectomy is a common ambulatory procedure typically performed for recurrent tonsillitis and obstructive sleep apnea.^1-6^ Other indications include tonsillar stones and recurrent peritonsillar abscesses.^3-6^ Malignancy or asymmetric tonsils are also indications, more common in the adult population.^3-6^

The tonsillar bed is highly vascular and as such, post-tonsillectomy hemorrhage is a well-known complication.^7^ Rates of post-tonsillectomy hemorrhage vary, and severe bleeds can be fatal.^7^ Death is rare, but unfortunately can still occur as has been recently highlighted within our nation.^8^ Other complications include decreased oral intake, dehydration, pain, nausea, and vomiting.^3,9-11^ These complications can result in emergency department (ED) visits and readmissions, which contribute to the burden on overstrained health services, specifically EDs.^3,9,11,12^ Post-tonsillectomy patients are reported to have the highest rates of ED use among ambulatory surgeries in Ontario, Canada, and these presentations increase costs to healthcare systems.^13,14^

Understanding and reducing postoperative ED visits is one strategy to relieve the strain on EDs, lower healthcare costs, and improve patient outcomes. Previous research has focused on post-tonsillectomy bleeds and pediatric populations.^14-16^ Few studies specifically describe ED usage following adult tonsillectomy; those that do are based in the United States.^14^ This topic is important in publicly funded healthcare systems, which may provide unique insights.

This is the first study to examine adult post-tonsillectomy ED visits in Canada’s publicly funded healthcare system. The objective of this study is to describe the rate and reason for ED visits within 90-days post-tonsillectomy in adult patients in the province of Nova Scotia.

## Methods

### Study Design

A retrospective cohort study was performed through chart review, assessing ED visits 90-days post-discharge from adult tonsillectomy in Nova Scotia’s Central Zone. Nova Scotia Central Zone houses the tertiary referral center for Atlantic Canada and has a regional catchment area of >500,000 patients.^17,18^ This study received approvals and waiver of consent from the Nova Scotia Health (NSH) Quality Improvement Office and the NSH Ethics Office as exempt from the Research Ethics Board review to allow collection, publication, and presentation of data (#1028137).

### Inclusion/Exclusion Criteria

All adult patients (≥16 years old) who underwent a primary procedure of tonsillectomy in Nova Scotia Central Zone, regardless of indication, with an ED visit anywhere in the province of Nova Scotia (not exclusive to Central Zone) within 90-days post-discharge were included. Surgery inclusion dates were April 1, 2016 to March 31, 2022, with the ED visit dates extended from April 1, 2016 to June 30, 2022, to allow a 90-days post-discharge window. Tonsillectomy patients were identified using NSH administrative datasets Discharge Abstract Database (DAD) and the National Ambulatory Care Reporting System (NACRS) who had a primary procedure Canadian Classification of Health Intervention (CCI) code of 1FR89LA; 1FR89WJ; 1FR87LA; 1FR59JAGX; 1FR78DAAB.^19^ Only patients with a valid Nova Scotia Health card were included in this study, as out-of-province residents were more likely to visit an ED in their home province. Patients who underwent laser/robotic procedures or tonsillectomy with resection of other oropharyngeal subsites were excluded. Patients aged ≥16 years old receive care within the adult hospital system, rather than the designated pediatric hospital in Nova Scotia. Therefore, an age cutoff of ≥16 years old was selected to provide a thorough review of tonsillectomy cases within the adult hospital system.

### Data Collection

A standardized web platform data collection form was developed in Research Electronic Data Capture (REDCap) before beginning chart reviews.^20,21^ Administrative hospital data contributed patient age, sex, primary care status listed on the ED chart, and Charlson comorbidity score to demographically describe the cohort.^22^ Patient’s residence categorization of urban or rural was determined by Canada Posts Corporation (CPC) postal code designation of second digit equal to zero classified as “rural.”^23^ The date of the procedure, site of ED visit, and indication for surgery were also sourced from hospital administrative health data.

To understand the nature of the ED visit, the triage chief complaint, discharge diagnosis, discharge location (admission or home), and length of stay in the ED were collected. It was also recorded if the visit was *surgical* or *medical*: surgical visits were defined as any visits related to the surgery, while all other visits were defined as medical. If a patient had multiple symptoms/reasons for presentation to the ED, the most concerning was selected as the reason for presentation (ie, if a patient presented with a concern for bleeding and pain, bleeding was selected as the reason for presentation as this is the more concerning feature). Surgical ED visits that did not result in admission and were not due to a presentation of bleeding were deemed “potentially preventable.” Presentations for bleeding were subclassified into primary bleeding (0-1 day post-surgery) and secondary bleeding (>1 day post-surgery).

### Data Analysis

The rate of presentation post-tonsillectomy and the prevalence of presenting concerns were determined. The results were reported descriptively with categorical variables presented as absolute counts with percentages and central tendencies expressed as means ± standard deviations (SD) for continuous variables and median with interquartile range (IQR) for non-normalized ED length of stay.

**Figure 1.**
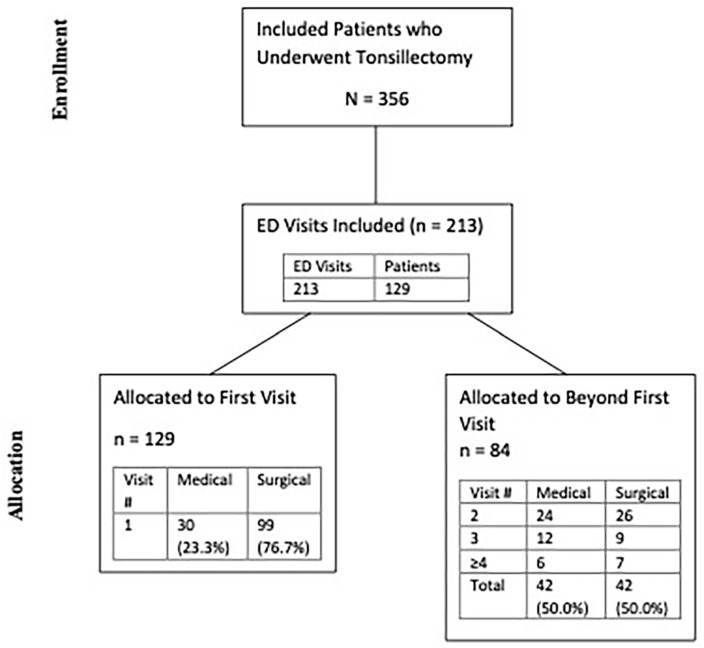
Patient flow based on ED visits. Abbreviations: ED, Emergency Department.

The ED presentations were separated into first ED visits and repeat ED visits (Figure 1). ED visit rates were calculated based on first ED visits to not skew results due to repeat visits for the same presentation/patient. Results with values N < 5 were suppressed and listed as **N < 5 as per our approval requirements for privacy.

## Results

### Overall ED Visits

During the 6-year study period, adult tonsillectomies meeting our inclusion criteria were performed on 356 patients in Nova Scotia Central Zone. A total of 213 ED visits for any presentation from 129 patients occurred within 90-days post-tonsillectomy discharge, representing a 36.2% ED visit rate regardless of presentation (Figure 1). Less than five patients were excluded for being laser/robotic cases or involving resection of other oropharyngeal subsites.

### Demographics, Surgical Technique, & Indications

Table 1 highlights the demographics of the patients with ED visits within 90-days post-tonsillectomy. The mean age of the cohort was 28.1 ± 13.8 years. Most patients were female (95/129; 73.6%), lived in urban locations (108/129; 83.7%), and had a primary care provider listed (119/129; 92.2%).

**Table 1.** Patient Demographics.

	1-7 days	8-30 days	31-90 days
Characteristic	ED Visit, N (%)	ED Visit N (%)	ED Visit N (%)
Total Patients	86 (66.7)	19 (14.7)	24 (18.6)
Age mean ± SD (yrs)	25.4 ± 10.4	31.8 ± 14.3	34.9 ± 20.4
Gender			
Female	64 (74.4)	15 (79.0)	16 (66.7)
Male	22 (25.6)	4 (21.1)	8 (33.3)
Patient geography			
Rural	12 (13.9)	3 (15.8)	6 (25.0)
Urban	74 (86.1)	16 (84.2)	18 (75.0)
Primary care status			
Has primary care	79 (91.9)	18 (94.7)	22 (91.7)
No primary care	7 (8.1)	1 (5.3)	2 (8.3)
Comorbidity score			
0	82 (93.4)	17 (89.5)	16 (66.7)
1	**N < 5	**N < 5	**N < 5
2	**N < 5	**N < 5	**N < 5
3	**N < 5	**N < 5	**N < 5
5	**N < 5	**N < 5	**N < 5
10	**N < 5	**N < 5	**N < 5
Type of visit			
Medical	**N < 5	5 (26.3)	23 (95.8)
Surgical	84 (97.7)	14 (73.7)	1 (4.2)
Days between discharge and ED visit, mean ± SD	3.1 ± 2.0	13.0 ± 5.7	60.4 ± 17.7
Hours in ED, median (IQR)	3.7 (2.2-5.1)*	3.9 (2.5-5.6)	3.4 (2.2-5.6)
Total hours	321	105	136
Medical	10	47	134
Surgical	311	58	2

Abbreviations: ED, Emergency Department; SD, standard deviation; yrs: years; **cell suppression for counts <5 as per approval regulations; *data from one patient missing.

This study screened for five surgical CCI procedure codes, reducing to four with associated ED visits. The most common is total tonsillectomy, 85.3%, with the remaining being total tonsillectomy and adenoidectomy, or destruction/excision of tonsils and adenoids (**N < 5).

### ED Presentation Characteristics

**Figure 2.**
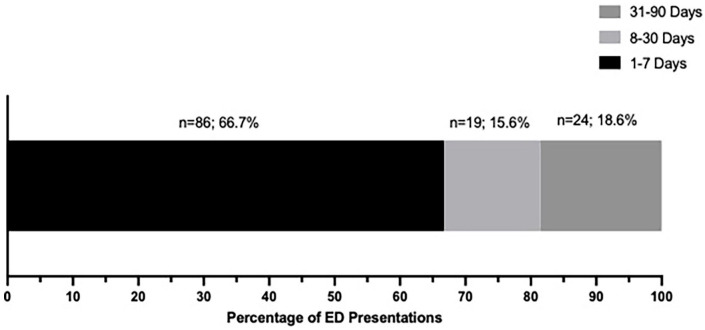
Proportion of days between discharge and initial ED visit. Abbreviations: ED, Emergency Department.

Of the 129 first ED presentations within 90-days post-tonsillectomy, 99 (76.7%) were deemed to be related to their tonsillectomy, representing a surgical ED rebound rate of 27.8% (99/356) (Figure 1). Most ED visits occurred within the first 1 to 7 days post-discharge (77/129; 81.4%), and 86/129 (66.7%) occurred within the first 7 days (Figure 2/Table 1). Most patients rebounded to an ED within the Nova Scotia Central Zone (105/129; 81.4%). The most common surgical indication among the patients with an ED visit was infectious (105/129; 81.4%) (Table 2).

**Table 2.** Indications for Initial Tonsillectomy among Patients who Rebounded to the Emergency Department.

Indication	N (%)
Infectious (Tonsillitis/Pharyngitis)	105 (81.4)
Sleep Apnea/Tonsillar Hypertrophy	14 (10.9)
Malignancy	5 (3.9)
Other	5 (3.9)

### Surgical ED Presentations

**Figure 3.**
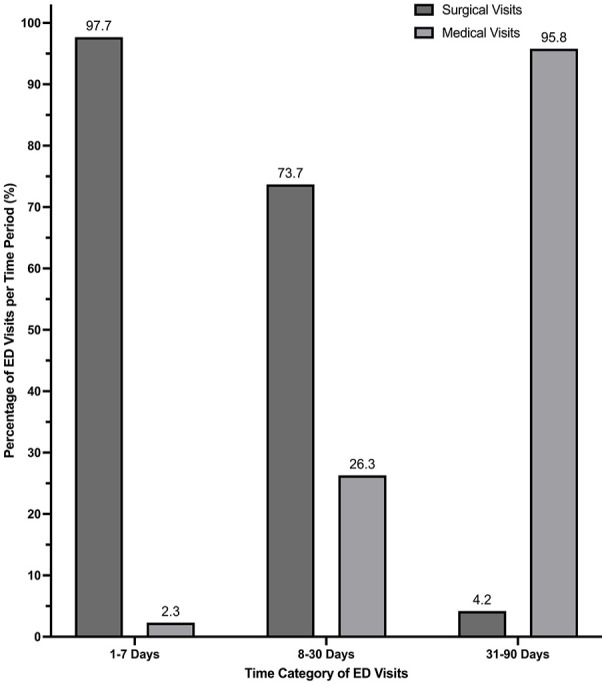
Percentage of ED visits per post-discharge day period. Abbreviations: ED, Emergency Department.

Surgical visits accounted for 97.7% (84/86) of visits within 1 to 7 days post-discharge (Figure 3/Table 1). This distribution reversed during the 31 to 90-days period, with 95.8% of visits (23/24) being medical (Figure 3). Patients spent a median time of 3.6 (IQR 2.3-5.1) hours in the ED (Table 1). A total of 575.1 patient hours were spent in the ED post-tonsillectomy, with 384.6 hours related to surgical visits (Table 1).

The most common surgical reasons for presentation to the ED were bleeding (47/99; 47.5%) and pain (36/99; 36.4%) (Table 3). The majority of surgical visits were discharged home from the ED or left without being seen (72/99; 72.7%) (Table 4). Of the surgical visits, 27/99 (27.3%) were admitted to the hospital (Table 4). Of these surgical admissions, 22/27 (81.5%) were due to post-tonsillectomy bleeding. Most presentations of postoperative pain without bleeding were discharged home or left without being seen (33/36; 91.7%). Of all patients presenting without bleeding, 48/52 (92.3%) were discharged home or left without being seen. These visits were deemed “potentially preventable,” suggesting 48.5% of all surgical visits (n = 48/99) are potentially preventable.

**Table 3.** ED Presentation Reason.

ED presentation reason	Number of presentations; N (%)
Surgical visits (N = 99)	
Bleeding	47 (47.5)
Primary bleeds (0-1 days post-surgery)	14 (29.8)
Secondary bleeds (>1 day post-surgery)	33 (70.2)
Pain	36 (36.4)
Other related to surgery	7 (7.1)
Nausea/Vomiting	6 (6.1)
Infection	**N < 5
Breathing difficulties	**N < 5
Diarrhea/Constipation	**N < 5
Medical Visits (N = 30)	
Other medical not related to surgery	22 (73.3)
Pain unrelated to surgery	6 (20.0)
Cardiovascular not related to surgery	**N < 5
Endocrine not related to surgery	**N < 5
Fever of unknown origin	**N < 5
Musculoskeletal not related to surgery	**N < 5

Abbreviations: ED, Emergency Department; **cell suppression for counts <5 as per approval regulations.

% is reported is of surgical visits and medical visits separately.

**Table 4.** ED Disposition.

Disposition	Medical and surgical visits, N (%)
Medical and surgical visits (N = 129)	
Discharged home	88 (68.2)
Admitted to hospital	30 (23.3)
Left without being seen	11 (8.5)
Surgical visits only (N = 99)	
Discharged home	67 (67.7)
Admitted to hospital	27 (27.3)
Left without being seen	5 (5.1)

In total, 13.2% (47/356) of all patients rebounded due to a concern of bleeding. Further, 6.2% (22/356) of all patients were admitted to the hospital for observation due to bleeding. Return to the OR for bleeding was required for <1.5% (<5/356) of all patients. Secondary bleeding represented 70.2% of all bleeding presentations (33/47) (Table 3). Primary bleeds represented 29.8% of presentations (14/47) (Table 3).

## Discussion

ED visits following common surgeries may lead to increased burden on healthcare systems, while negatively impacting patient experiences. It is valuable to assess ED visits postoperatively to identify areas for quality improvement. This is the first study examining ED visits within 90-days post-adult tonsillectomy in Canada’s publicly funded health system. This 6-year cohort showed a surgery-related ED rebound rate of 27.8%. The most frequent surgical reasons for ED presentation were bleeding (47.5%) and pain (36.4%). Most surgical patients were discharged home directly from the ED or left without being seen (n = 73; 73.7%). Half of all surgical visits were deemed “potentially preventable.”

Overall, bleeding was the most common reason for ED presentation and hospital admission. Previous studies looking at ED visits post-tonsillectomy have also reported bleeding or pain to be the most common presentations.^3,5-7,9-11,14-16^ In this cohort, 13.2% of patients rebounded to the ED for concern of bleeding, 6.2% of patients were admitted for observation due to bleeding, and less than 1.5% required a return to the operating room. This rate of ED presentation for post-tonsillectomy bleeding is higher than expected compared to pediatric populations, but is in line with recent literature in the adult population.^3,5,6,11,14-16^ A 2022 study in American adults showed a 13.1% post-tonsillectomy bleed rate.^3^ At our center, patients are advised to seek urgent care if there are any signs of bleeding in the postoperative period. This may contribute to higher ED rebound rates for bleeding concerns. However, given the potentially fatal risk of post-tonsillectomy hemorrhage, it is felt that a low threshold for patients to seek care in the ED for this presentation is prudent. For this reason, these visits were not considered “potentially preventable.”

Most post-tonsillectomy bleeding presentations were considered secondary bleeds (70.2%). This is consistent with previous literature in the adult population that showed 88.7% of bleeds being secondary in nature.^24^ However, the overall rate of bleeding in this study was higher than our study at 21.8% vs. 13.2%.^24^ Lindquist et al. did not compare pediatric to adult patients; however, they did find that older pediatric patients, specifically those over the age of 6 years, were at significantly higher risk of post-tonsillectomy bleeding and reoperation to control the bleeding.^25^ Similarly, Kim et al. found that patients older than 15 years had higher rates of post-tonsillectomy bleeding and lower rates of spontaneous cessation, resulting in more frequent operative management.^26^ Lee et al. also found that patients ≥ 12 years old had a significantly higher rate of post-tonsillectomy bleeding.^27^ The overall higher rate of bleeding in adult compared to pediatric populations helps explain how half (47%) of the surgical ED visits in this patient cohort were due to a concern for bleeding.

Pain related to surgery was the second most common ED presentation. Most patients were treated symptomatically in the ED and discharged home or left without being seen. Based on these results, reducing ED presentations secondary to post-tonsillectomy pain would be an effective quality improvement target. Pain is a well-known sequela following tonsillectomy, which can result in odynophagia and dehydration, worsening a patient’s pain. Perioperative counseling regarding pain control and hydration is crucial to post-tonsillectomy care. Appropriately dosed opioids, along with acetaminophen and nonsteroidal anti-inflammatory medications, are used for pain control post-tonsillectomy and are standard of practice at our center ^28^ However, postoperative opioid prescribing patterns among otolaryngologists show high variability.^28-30^ Standardization and optimization of opioid/analgesia prescribing patterns and patient education post-tonsillectomy may reduce healthcare utilization and improve patient satisfaction.

Previous literature has looked at reducing post-tonsillectomy pain-related ED visits. Wozney et al. implemented a text messaging service “Tonsil-Text-To-Me,” which was an automated SMS texting service that would send time-sensitive educational materials and activity reminders to caregivers post-pediatric tonsillectomy.^31^ This intervention was shown to be effective in reducing postoperative pain and improving caregiver satisfaction.^31^ Dexamethasone at the time of operation and postoperatively may also be effective at reducing postoperative pain.^32-34^ In addition, studies have also demonstrated various substances (eg, honey) and tactics (eg, cooling of tonsillar fossa) may reduce postoperative pain.^35-37^ Jain et al. found preoperative education feasible, yet ineffective in reducing ED presentations post-tonsillectomy, which may be attributable to poor patient recall of in-person verbal instructions.^38-40^ However, proper patient education to ensure appropriate pain control upon discharge is crucial, and further strategies warrant investigation.

Although post-tonsillectomy hemorrhage requires cautious and urgent management, which is best facilitated through the ED, utilizing outpatient otolaryngology and primary care clinics may reduce ED utilization for other common post-tonsillectomy presentations, such as pain. This may also improve patient satisfaction, given that the median time spent in the ED for patients in our cohort was 3.6 hours, with patients spending 384.6 hours in the ED for surgical presentations. Most patients (92.2%) in our cohort had a primary care provider listed, yet presented to the ED. Previous reports have shown that Canadians with family physicians report good access to their providers.^41^ This may represent uncertainty or discomfort among primary care providers in managing postoperative concerns where collaboration with surgical services may enhance comfort and education. It may also represent patient perceptions of requiring an ED visit for postoperative concerns or issues with access to priority primary care within our region. Also, when complications arise after hours, visits to the ED may be patients’ only point of access to urgent medical care. Regardless, this burden should not solely fall on the shoulders of primary care or the ED. Access to otolaryngology advice or a rapid access clinic for management and education around postoperative pain and other routine complications would likely facilitate the reduction of ED use and enhance patient care. Ultimately, this cohort suggests half of surgical ED visits may be “preventable” and strategies to improve education and access to care would facilitate the off-loading of EDs.

The “potentially preventable” surgical ED visits included pain without bleeding, which may be avoided with improved patient education and better pain control. However, this category also included nausea and vomiting, infection, breathing difficulties, and diarrhea/constipation. These were included as “potentially preventable” visits only if they were discharged home directly from the ED. Those who were admitted were not considered “potentially preventable” as they required either observation or admission for symptomatic management. For those who did not require admission, the review of the ED chart often showed the use of over-the-counter medication and education before discharge home. It is felt that these visits may be potentially preventable. However, it is challenging to ensure this based on retrospective chart review, which is an inherent limitation of this study, specific to this subset of patients.

Overall, a quarter of patients presented to the ED with surgical presentations within 90-days post-tonsillectomy discharge in our study. This rate is higher than initially expected compared to both previous adult and pediatric cohorts. Upon review of recent literature on adult and pediatric post-tonsillectomy complications, presentation rates were noted to be in the ~10% to 20% range.^14,16,38,42^ Specifically, a large cohort study based out of the United States reported a 21.5% complication rate post-adult tonsillectomy, but only a 10.7% ED visit rate within 30-days post-procedure.^14^ Ontario data analyzed common ambulatory procedures from numerous specialties and found tonsillectomy to have the highest rate of ED usage within 3 days post-procedure at 8.1%, which was almost double the next highest procedure (cholecystectomy 4.2%).^13^ Studies before 2014 focused mostly on bleed rates, reoperations, and major complications, potentially downplaying re-visits due to pain and dehydration. Further, in comparison to larger database studies, this cohort underwent independent chart review. Lack of formal chart review in larger studies may inadvertently exclude ED visits that seem unrelated to the operation based on oversights in coding. The higher rate in this study may also be due to the publicly funded health system in Canada reducing financial barriers to ED presentation post-tonsillectomy, subsequently resulting in a “lower threshold” to seek acute care. Moreover, seeking outpatient care after hours is a known challenge in our community and may result in increased presentation to EDs. This study also extended the inclusion period to 90-days post-tonsillectomy compared to <30 days in previous studies.^13,14^ This may impact the ED rebound rate as 24 patients presented to the ED within 31 to 90 days postoperatively. However, this had a limited impact on the surgical ED rebound rate, as only one of these visits was considered surgical in nature (Table 1). Finally, this cohort also included patients who left without being seen after being triaged in the ED. All of these factors may have contributed to higher ED rebound rates.

The higher rate of ED rebound may also suggest a higher rate of complications among adult tonsillectomy patients as compared to their pediatric counterparts. Although data is limited, one study found pain and bleeding were significantly more common in adults compared to pediatric patients post-tonsillectomy.^39^ However, this study was limited to 178 pediatric tonsillectomies and 35 adult tonsillectomies. Further, as mentioned earlier in the discussion, previous literature suggests that older patients are more prone to post-tonsillectomy bleeding and are more likely to require operative intervention compared to their younger counterparts.^24-27^ This higher rate of bleeding may be enough to account for more ED visits in adults compared to pediatric populations. In this cohort, half (47%) of all surgical ED visits were due to a concern for bleeding. However, the lack of clarity in this area may stem from a relative shortage of data in adults compared to children. The current data may be an avenue to improve information transfer and complication rates quoted to adult patients preoperatively. Further, it may represent a path for optimization of postoperative pain control and patient education.

This study provides a comprehensive review of all ED visits 90-days post-discharge from adult tonsillectomy performed in Nova Scotia Central Zone. All ED visits from across the province for these patients were captured. However, our study was retrospective in nature; prospective collection of data may provide greater insights, reduce biases, and facilitate the trial of novel interventions. We also included all indications for tonsillectomy, which provides a larger sample size. Previous studies, however, have used stricter inclusion and exclusion criteria, which should be considered when interpreting the current results. Further, we were unable to stratify risk factors or patient demographics, such as geography, as predictive of ED usage due to REB limitations restricting access to demographic data points to only those patients who presented to an ED, not those who were operated on and did not present to an ED. Finally, we did not have access to patients who were seen in outpatient otolaryngology or primary care clinics, potentially underestimating our complication rates and the utilization of these resources in the postoperative period. Nevertheless, we feel this study provides unique insights into post-tonsillectomy ED utilization. Future areas of study will include improving postoperative outpatient otolaryngology access as well as analyzing prescribing patterns, patient characteristics, and surgical factors to determine if these contribute to postoperative ED visits.

## Conclusion

We present the first study assessing post-adult tonsillectomy ED utilization in Canada’s publicly funded healthcare system. The surgical ED rebound rate was 27.8%, adding to recent literature suggesting higher rates of complications post-adult tonsillectomy, with most visits due to bleeding and/or pain. Half of all surgical visits were deemed to be potentially preventable. These results provide greater insights into preoperative counseling and education of adult tonsillectomy patients while emphasizing the optimization of postoperative pain control to reduce ED visits.
